# Diseases of the Nose and Throat: A Text-Book for Students and Practitioners

**Published:** 1916-12

**Authors:** 


**Diseases of the Nose and Throat: A Text-book for Students and Practitioners.** By Sir St. Clair Thomson, M.D. Second Edition. Pp. xvi. 858. London: Cassell & Company Ltd. 1916.

The second edition of this excellent and very complete treatise on diseases of the nose and throat, coming so relatively shortly after the first edition, which we reviewed at length, calls for no extended notice. But several of the sections have been largely revised and re-written, while an entirely new section has been written on intranasal dacryocystotomy, and one on the nasal route to pituitary tumours. Amid the wealth of information afforded, we were disappointed to find no adequate reference to endo-rhinoscopic methods, without which many cases of nasal catarrh remain obscure, though so easy to detect and elucidate by means of the "nasal periscope." Further, some of the more recently-developed diagnostic methods, and endonasal operative measures, with which we have been familiar for some years, receive scant notice. For the wealth of illustration, both coloured and black and white, we have nothing but praise.

